# Supplementary material for: An unconstrained approach to systematic structural and energetic screening of materials interfaces
Source: Nat Commun. 2022 Oct 20;13:6236. doi: 10.1038/s41467-022-33414-6 (PMC9585034; doi:10.1038/s41467-022-33414-6)
Supplement: Supplementary file 1 — Supplementary Info [file 41467_2022_33414_MOESM1_ESM.pdf]

Supplementary Information

An Unconstrained Approach to Systematic Structural and Energetic Screening of Materials Interfaces, Di Liberto et al.

Supplementary note 1

The E_{sep} energy values at each point **s** = (s_x,s_y) of our reported relative interface energy displacement maps have been refined by performing two complementary scans. First, we displace the first disk with the respect to the second by positive vectors (s_x,s_y) and evaluated $E_{sep1}(s_x,s_y)$, at each point. We then performed a second scan by displacing the second disk with respect to the first one by the complementary vectors ($-s_x$,$-s_y$) and evaluated $E_{sep2}(-s_x,-s_y)$. For an infinite model, the two pictures are the same and $E_{sep1}(s_x,s_y) = E_{sep2}(-s_x,-s_y)$ For finite systems, this symmetry is broken, and for every energy in the scan we have: $E_{spe1} – E_{sep2} = \Delta E$. As both E_{sep1} and E_{spe2} are equally valid finite estimates of the actual interface energy for any displacement we correct both E_{sep1} and E_{sep2} towards the mean of the difference to define a refined scan of displacement-dependent energies: $E_{refined} = E_{sep1} + \delta = E_{sep2} - \delta$, where $\delta = \Delta E / 2$. Supplementary figure 1 shows the raw E_{sep1} scan for anatase/anatase at α = 0° (left) together with the refined scan (right). Compared to the original scan, the refined scan has smoother variations in energy due to inter-basin regions being filled in by δ and has some minor induced translational asymmetries. Overall, the refined scan retains the overall pattern of maxima and minima of the original scan, where the relative interface energies of important maxima and minima are relatively unchanged (typically with energy difference of <0.03 J/m^2).

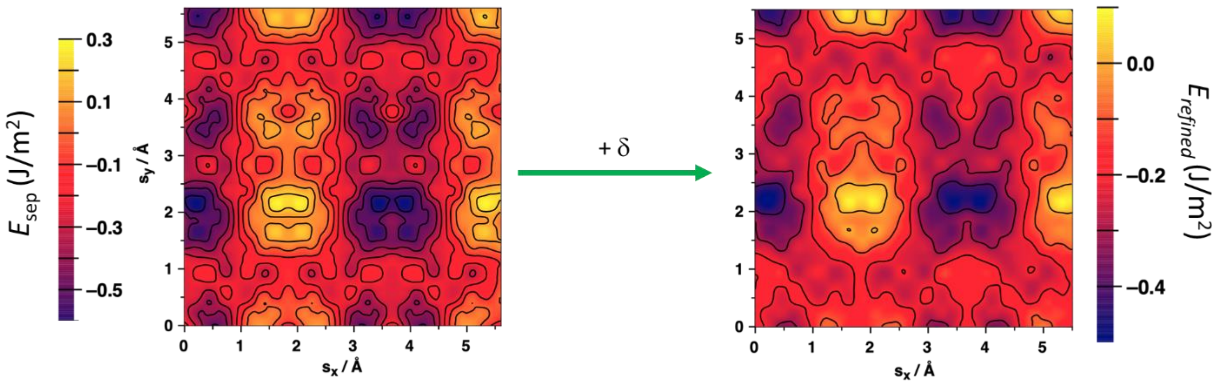

Supplementary figure 1: **Energy scan refinement due to finite size effects.** Raw E_{sep} displacement scan of the (101)/(001) interface for α = 0° (left) and the corresponding finite size refined scan (right).

Supplementary note 2

The work of separation, E_{sep}, is used throughout to calculate relative interface energies and is given by:

$$E_{sep} = (E_{tot} - E_{surf1} - E_{surf2}) / 2A,$$

where E_{tot} is the total energy of the double disk system, E_{surf1} and E_{surf2} are the energies of the two separate disks, and A is the interfacial area of the two interacting disks. E_{sep} for a double disk model of the (001)/(101) interface is found to be adequately converged for a disk radius of 2.5 nm (see Supplementary figure 2).

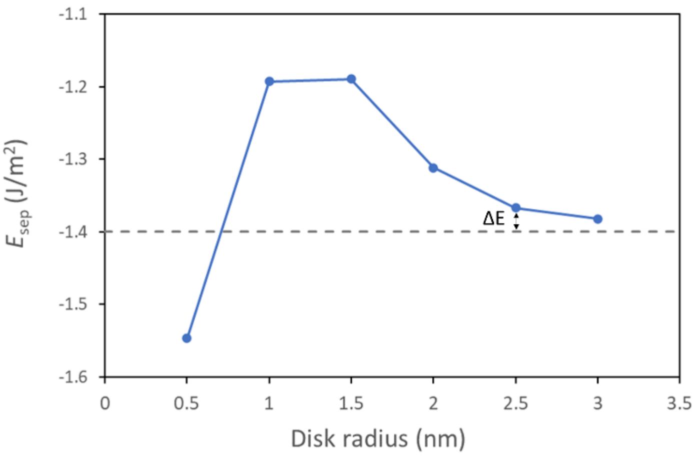

Supplementary figure 2. Convergence of E_{sep} with increasing disk radius for the (001)/(101) interface. The dashed grey line indicates the estimated limiting interface energy for an infinite system. The corresponding finite size interface energy error (ΔE) when using disks of 2.5 nm radius is 0.03 J/m^2.

Supplementary note 3

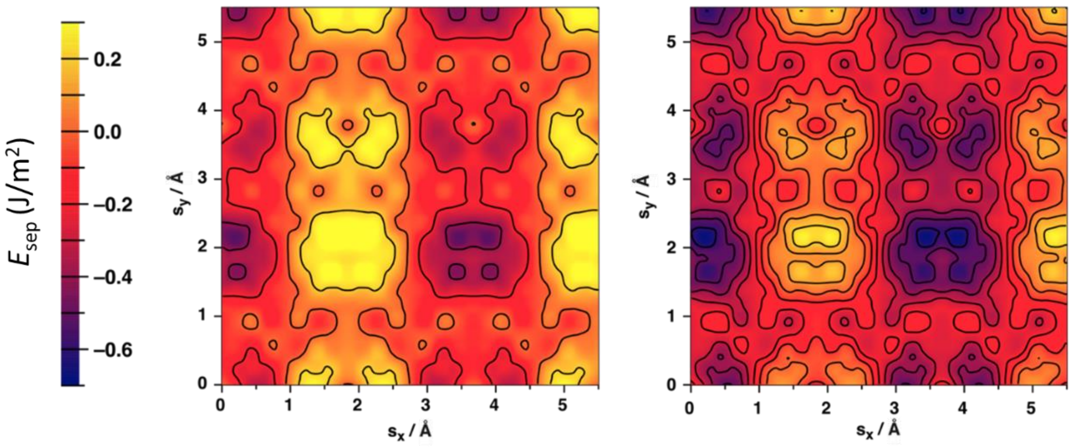

Supplementary figure 3. Convergence of interface energy with degree of relaxation. Relative interface stability displacement maps for α = 0° for non-relaxed disks (left) and using an inner relaxed atom region of 1.7 nm radius (right) for the (001)/(101) interface.

In Supplementary figure 3 we compare a displacement scan at α = 0° calculated when using: i) our adopted relaxation protocol (i.e. atoms relaxed within a radius of 1.7 nm and thickness of 1 nm with all outer atoms fixed to bulk positions), and ii) disks with all atoms fixed to bulk position. Clearly, allowing inner interfacial region to relax slightly lowers the calculated energy in a systematic way.

To show this effect more clearly, we take three characteristic points from these maps, corresponding to: an E_{sep} maximum (max), E_{sep} minimum (min) and an intermediate E_{sep} between these values (between). We then follow the change in energy of each with respect to the percentage of the relaxed interfacial area (i.e. with respect to the maximum corresponding to the full 2.5 nm radius). We can see in each case that E_{sep} systematically decreases in a similar linear fashion with respect to the percentage of relaxed interfacial area employed. This implies that all relative interface energetic stabilities within a displacement scan are relatively unaffected. From this tendency we can also estimate that the E_{sep} values that we obtain using a relaxed region of 1.7 nm radius are systematically overestimated by ~0.2 J/m^2 relative to a fully relaxed system.

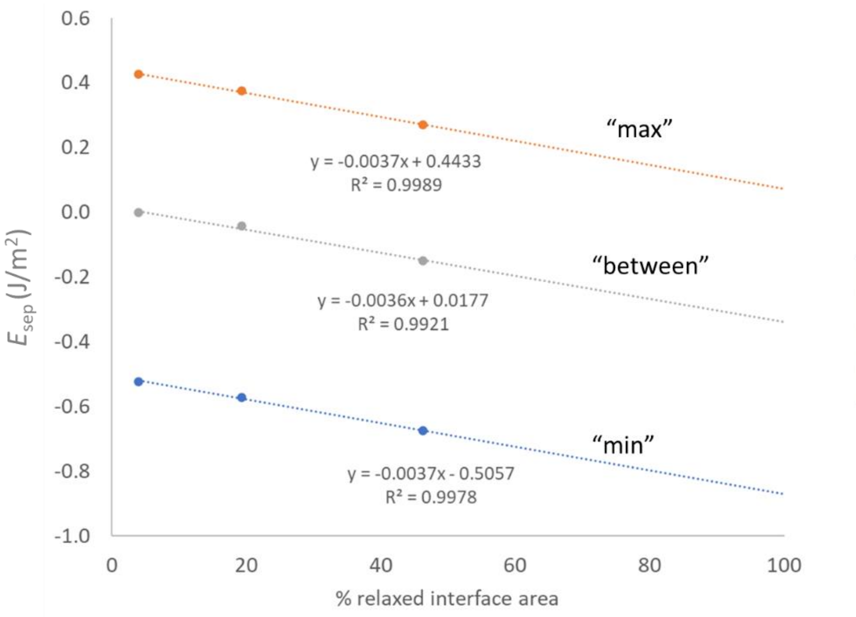

	46%	100%	ΔE
**max**	0.27	0.07	-0.20
**between**	-0.15	-0.34	-0.19
**min**	-0.68	-0.87	-0.20

Supplementary figure 4. Dependency of E_{sep} on size of relaxed disk region. Changes in calculated E_{sep} with respect to the percentage of area of the relaxed interfacial region for three interfaces taken from the maps in Supplementary figure 3 (left). Extrapolating from the fitted linear tendencies in each case we can estimate the E_{sep} values for the fully (100%) relaxed system (right).

Supplementary note 4

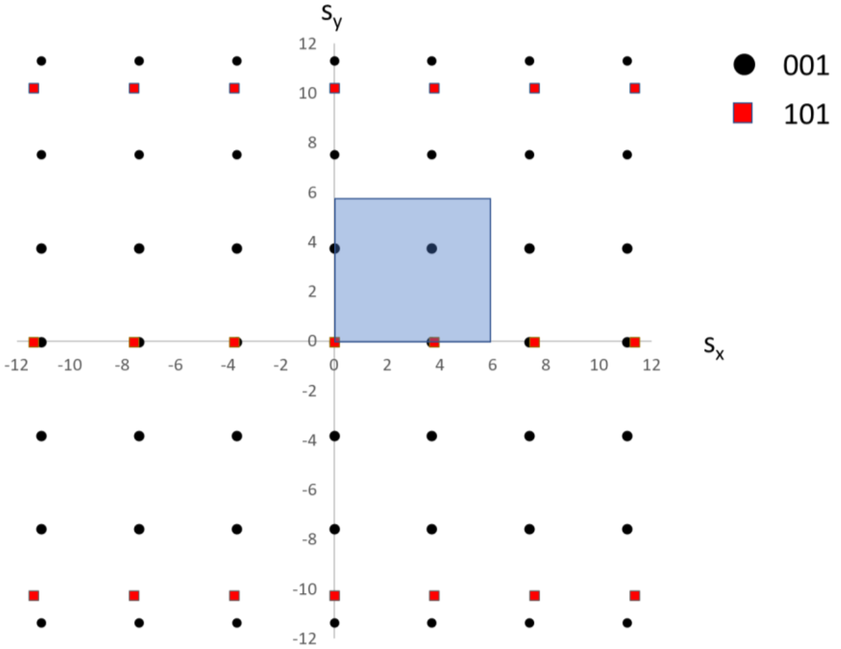

Supplementary figure 5. Equivalent anatase (001)/(101) interfaces. Symmetrically equivalent points with respect to displacements (Å) from an arbitrary initial interface configuration at **s** = (0,0) are shown with respect to (001) and (101) in-plane lattice parameters. The range of displacements sampled in the anatase (001)/(101) study is indicated by the blue shaded region (see Figure 1 in the main text for the corresponding E_{sep} displacement map).

Supplementary note 5

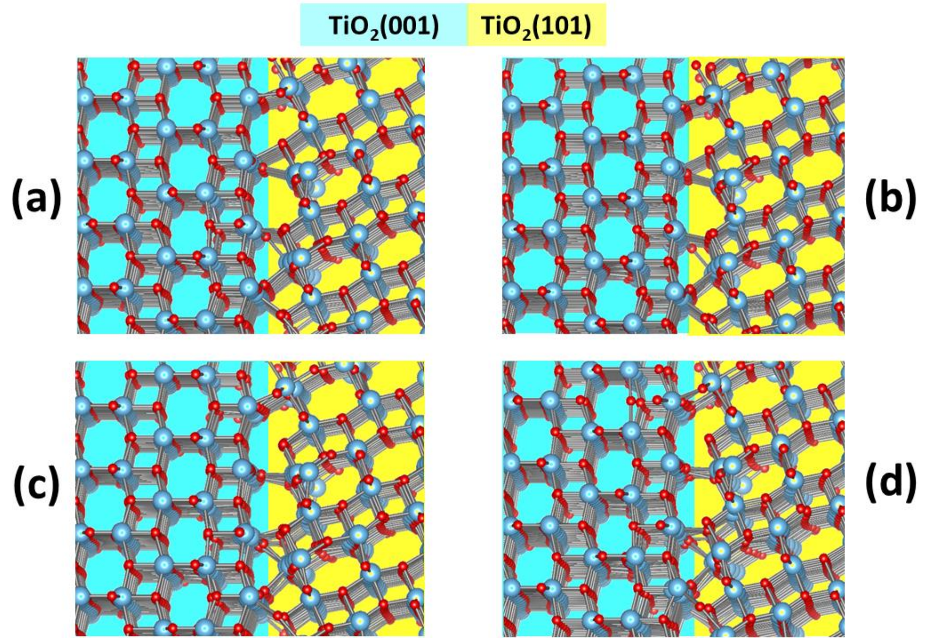

Supplementary figure 6: **Low energy (001)/(101) interfaces.** Structures of four low energy IP-optimised interfaces for α = 0° corresponding to the four near degenerate energy minima highlighted in Figure 2 in the main text (i.e. M1-M4). Cyan and yellow areas identify the (001) and (101) sides of the interface respectively. Atom key – Ti – blue, O – red.

Supplementary note 6

For any selected displacement we can perform a systematic twist angle scan. In the main text we use an angular increment of 5° for these scans. Below we show that the results are not significantly changed if we increase the resolution of the scan by five times by using a 1° increment indicating that 5° is sufficient for an initial twist angle scan.

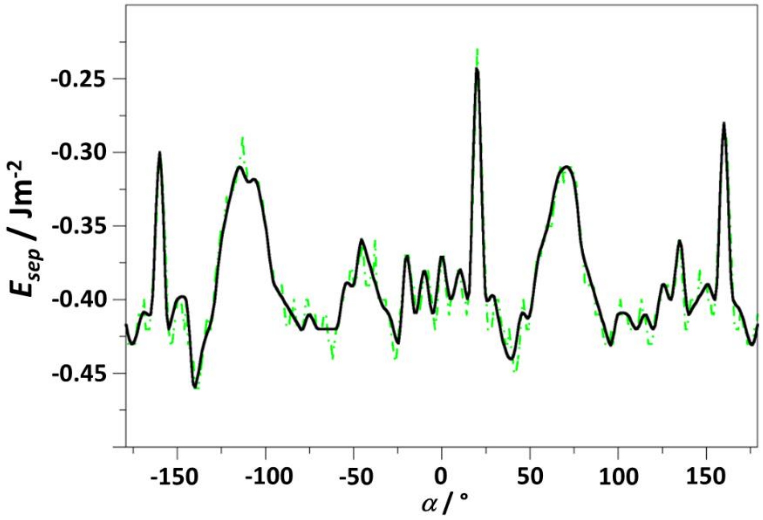

Supplementary figure 7. Effect of size of angular increment in twist angle scans. Comparison of calculated interface stability (E_{sep}) of the (001)/(101) anatase interface with respect to twist angle using an angular increment of 5° (solid black line) and 1° (dashed green line) for a fixed displacement.

Supplementary note 7

The calculated *a* and *c* lattice parameters obtained from an IP-based optimization of the anatase TiO_2 bulk crystal structure were found to be 3.770 and 9.570 Å respectively. The corresponding values predicted by periodic DFT calculations with the HSE06 functional were 3.775 and 9.478 Å, respectively. The IP-based and DFT based values are in good agreement with a deviation <1 % for both a and c. Both sets of values are also in good agreement with previously reported DFT data and experimental measurements, *a* = 3.785 and *c* = 9.514 Å. An IP versus DFT comparison of the structures of the optimised periodic (001) and (101) reference slabs cut from the anatase bulk crystal is provided in Supplementary tables 1 and 2.

System	*a* / Å	*b* / Å	*gamma* / °
TiO_2 (001) GULP	3.691	3.778	90
TiO_2 (001) DFT	3.616	3.846	90
Deviation %	2.0	1.8	0

Supplementary table 1: Calculated lattice parameters *a*, *b*, and *gamma* of TiO_2 (001) surfaces using IPs (GULP) and DFT/HSE06 (VASP). See main text for computational details.

System	*a* / Å	*b* / Å	*gamma* / °
TiO_2 (101) GULP	3.785	10.239	90
TiO_2 (101) DFT	3.644	10.295	90
Deviation %	3.9	0.5	0

Supplementary table 2: Calculated lattice parameters *a*, *b*, and *gamma* of TiO_2 (101) surfaces with interatomic potentials (GULP) and DFT/HSE06 (VASP). See main text for computational details.

The calculated lattice parameters of rutile (110) obtaining with our employed IPs are *a* = 3.013 Å and *b* = 6.337 Å, which are again in good agreement with the corresponding DFT results, 3.01 Å and 6.56 Å respectively. (e.g. see J. Phys. Chem. C 2017, 121, 28328−28338.).

Supplementary note 8

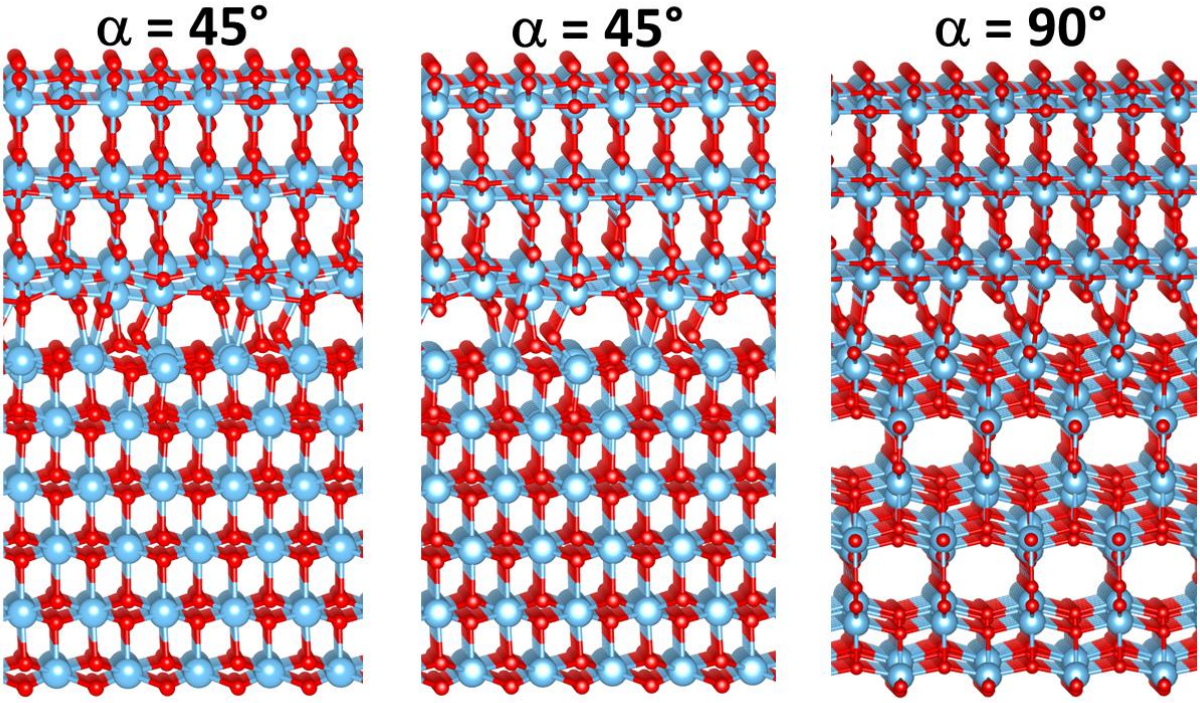

Supplementary figure 8. **Periodic DFT calculations of anatase (001)/(101) interfaces.** (001)/(101) interface structures obtained from periodic DFT/HSE06 optimisations in which the initial structures were, from the left to right: i) the minimum energy interface structure at α = 45° from our disk interface method, ii) an interface manually designed by rotating the (001) slab with respect to (101) by ~45° to ensure good cation-anion matching between the slabs and acceptable lattice mismatch (see ref. 31 in main text), iii) the minimum energy interface at α = 90° obtained from our disk interface screening method.

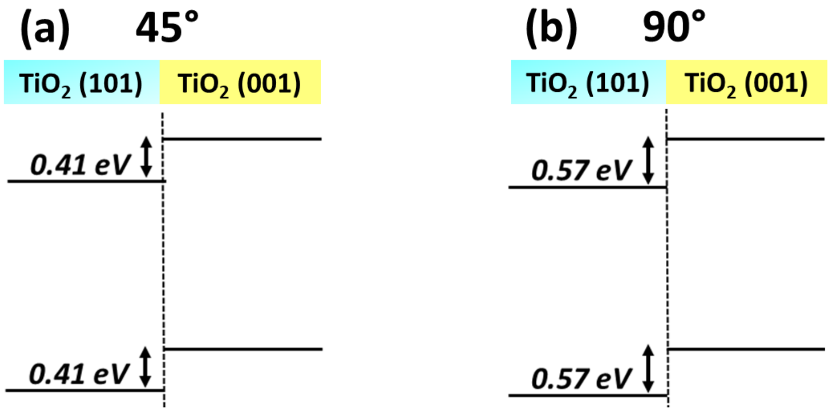

Supplementary figure 9. **Band edges at (001)/(101) interfaces.** Band edge alignment of anatase surface interface junction between (001) and (101) surfaces for: relative twist angles of: (a) 45° and (b) 90° (see structures in Supplementary figure 8).

In Supplementary figure 9 we show the calculated band offsets for the interfaces shown in Supplementary figure 8 (note that for α = 45° the two interfaces are essentially identical). The band offsets have been evaluated by using the potential line-up method. Namely, the plane-averaged electrostatic potential (*V*) has been evaluated for both interface and separated components. Then, the macroscopic average of *V*, can be used as an energy reference to align the band edges of separated components (see cited references in the main text for more details).

Supplementary note 9

The coincidence site lattice (CSL) approach is traditionally applied to grain boundaries (GBs) of densely packed monoelemental systems (typically metals). For a pure twist GB one can first align the lattice parameters of the two grains and then twist one lattice with respect to the other until one finds specific angles with periodic lattice site coincidences. Although CSL does not consider atoms, the idea is based on finding GBs with a good atomic fit and are which are thus expected to have higher energetic stabilities. CSL has often proven to be a useful geometric guide to low energy metal GBs.

In our work, we directly evaluate the relative energetic stabilities of interfaces between dissimilar surfaces of a semi-ionic compound in a non-periodic system (i.e. not a typical GB system). Simple geometric measures such as CSL for understanding interface stability have been reported as being lacking with respect to more direct atomistic models (e.g. "It is concluded that no general and useful criterion for low energy can be enshrined in a simple geometric framework. Any understanding of the variations of interfacial energy must take account of the atomic structure and the details of the bonding at the interface." – A. P. Sutton, R. W. Balluffi (1987), Overview no. 61: On geometric criteria for low interfacial energy, Acta Metallurgica, 35, 2177). In our study we wanted to use a CSL-type measure that was well tailored for our systems and thus less

susceptible to such criticism. In particular, the relatively high complexity of our systems means that a traditional CSL approach has limited applicability:

1.) When considering heterojunctions, traditional CSL does not give a unique prescription for finding the relative lattice displacement from where to start twisting. Therefore, we first perform a scan of rigid body displacements to obtain a low energy starting point for our twist angle scans. For consistency, we use these displacements for both our CS scans and our energy evaluations with respect to twist angle. Note that these displacements are determined independently of the underlying lattice sites for the respective surfaces.

2.) Traditional CSL is purely geometric and does not consider atoms nor discriminate between atom types/charges. Thus, CSLs for atomically heterogeneous systems can yield highly repulsive and energetically unstable interfaces. To take into account such cases, we give each atomic site a sign and a local spatial region (Gaussian distribution) and calculate the overlap between cation/anion sites in our coincidence measure. This means that we go beyond a purely lattice based approach to a measure which uses atoms positions when evaluating CSs. We note that CS approaches to rationalise interfaces using atoms positions are not new and were first proposed and used effectively in: Kronberg, M. L. and F. H. Wilson (1949), “Secondary recrystallization in copper”, Trans. Met. Soc. AIME, 185, 501.

Considering the above, to provide an atomic CS-based evaluation of our interfaces we assigned to each atom (*N*) having coordinates (x_0,y_0) at the interface between the two disks, an isotropic gaussian function of the type:

$$\varphi_N(x,y) = \frac{1}{\sqrt{\pi\sigma}} e^{-\frac{1}{2\sigma}[(x-x_0)^2+(y-y_0)^2]}$$

The Gaussian width (s) was set to be 0.1, which was found provide stable smoothing varying results. In this way, two atoms belonging to two different disks, but having the same *x* and *y* atomic coordinates at the crystallographic plane of the interface, would have the maximum overlap, calculated as:

$$I = \iint \varphi_M(x,y)\,\varphi_N(x,y)dxdy = \frac{1}{\pi\sigma}\iint e^{-\frac{1}{\sigma}[(x-x_0)^2+(y-y_0)^2]}dxdy = 1$$

We note that within this approach the positioning along the non-periodic direction *z* is not considered. In other words, this method provides most likely overlap atomic configurations in the plane defining the interface, and the *z*-coordinates will be given by the ideal atomic bond distances between the atoms belonging to the two disks.

In addition to simple atomic overlap, to account for the ionic nature of TiO_2 we use a signed overlap measure to account for cation-anion and cation-cation or anion-anion matching by assigning a positive or negative sign to atoms according to the sign of their formal charge. In such a scheme, a perfect overlap (coincidence) between two cations or two anions gives rise to a value of 1. Conversely, the perfect overlap

(coincidence) between a cation and an anion, results in an overlap equal to -1. Therefore, each possible overlap range can span from -1 to +1. For each applied displacement and rotation in our double disk scans we calculate the signed overlap integral for all atoms at the interface as:

$$I = \sum_{i=1}^{N_{(101)}} \sum_{j=1}^{M_{(001)}} I_{ij}.$$

Finally, we normalize the overall integral by the number of interface atoms to provide a final value between -1 and +1. The resulting signed atom-based CS maps are compared with the relative energy maps with respect to scanning a range of relative displacement and angles in Supplementary figure 10.

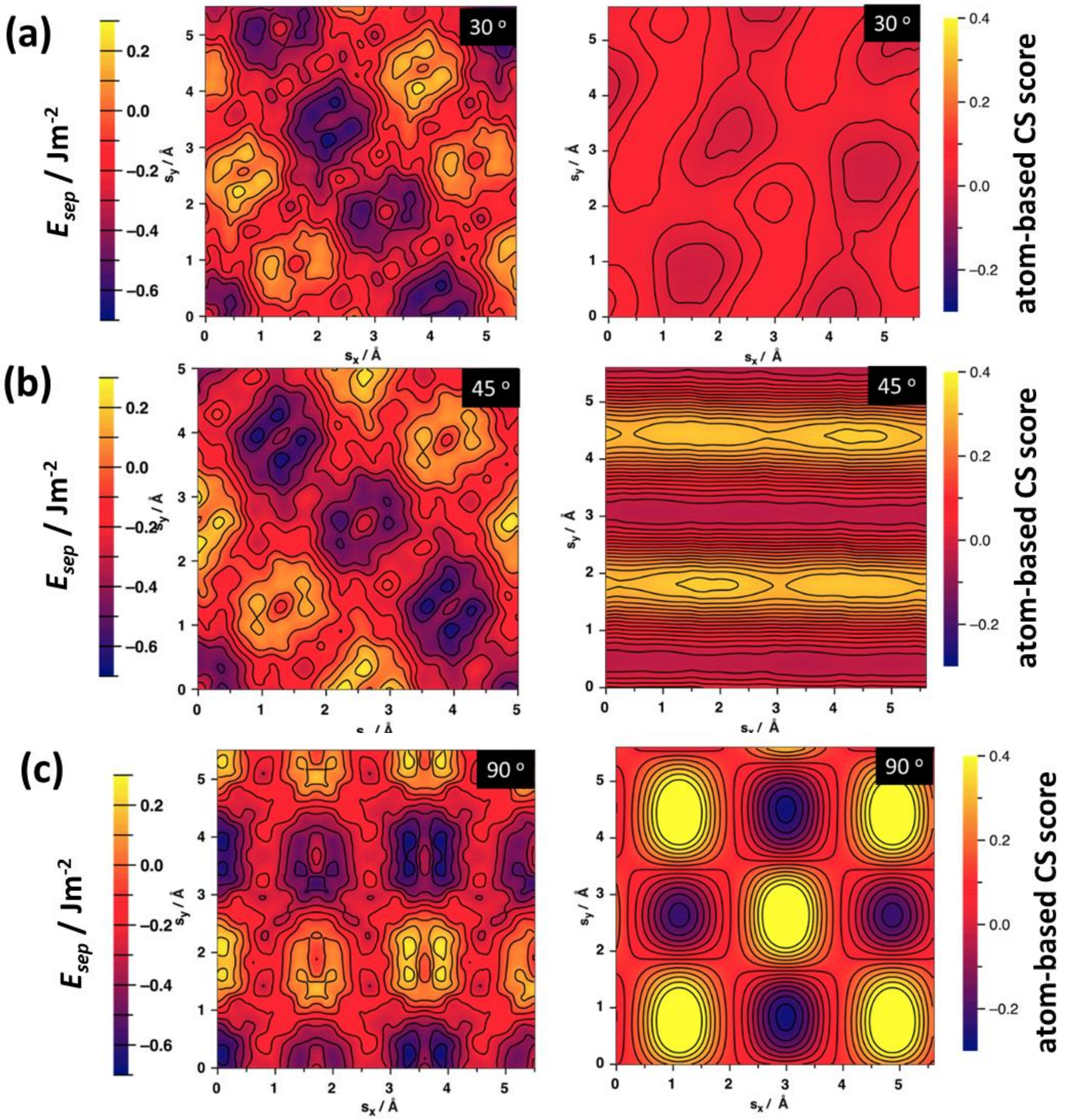

Supplementary figure 10: **Comparison of atom-based CS values and directly calculated energies for displacements.** Relative interface energy maps (left) versus signed atom-based CS maps (right) for the anatase (001)/(101) interface for relative in-plane displacements (s_x and s_y) and for α = 30° (a), 45° (b), and 90° (c). For the signed atom-based CS maps the highest cation-anion overlap is shown in yellow, while cation-cation or anion-anion overlap is indicated in blue.

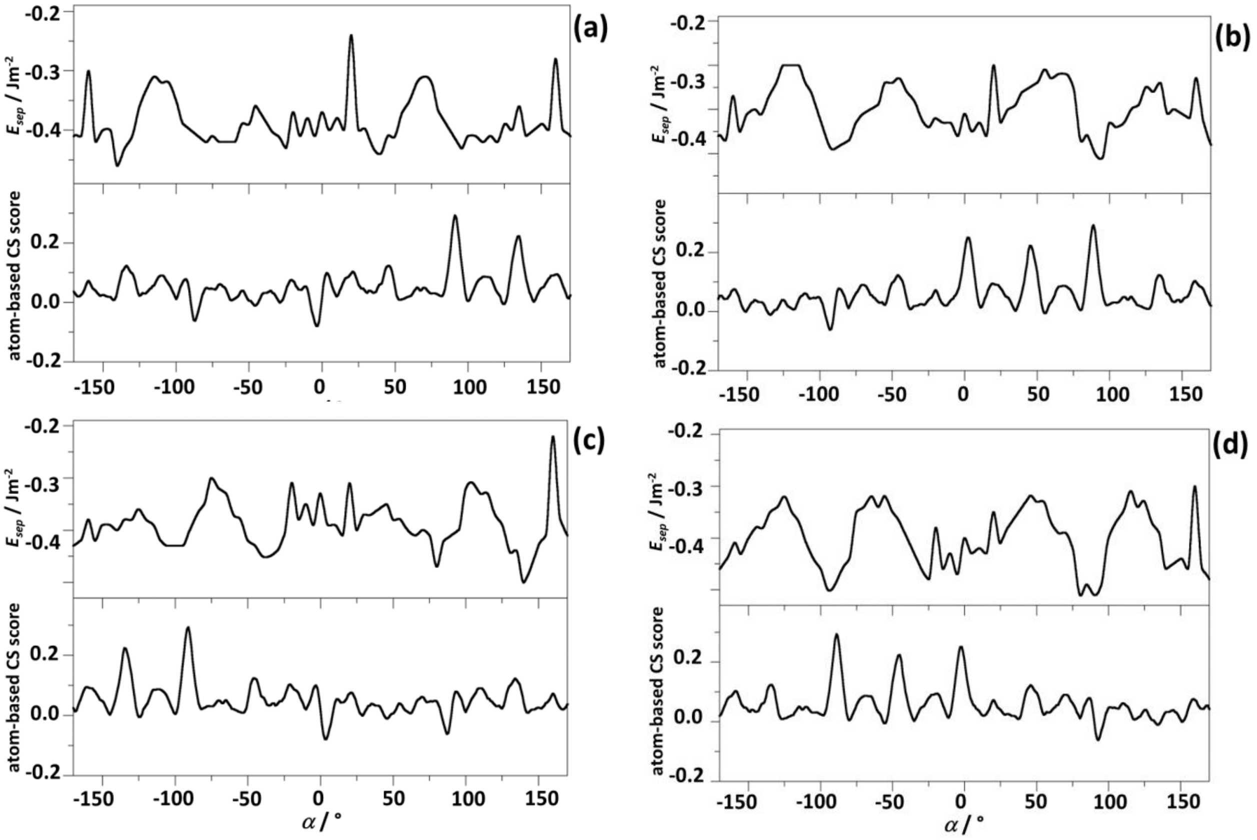

Supplementary figure 11. **Comparison of atom-based CS values and directly calculated energies for twist angles.** Angular relative interface energetic stability scans (top) and corresponding signed atom-based CS maps (bottom) for the anatase (001)/(101) interface with respect to four in-plane displacements (a), (b), (c), and (d) giving low energy structures (see figures 2 and 4 in the main text). For the signed atom-based CS maps cation-anion overlap leads to more positive values while cation-cation or anion-anion overlap leads to more negative values.

In addition to comparing displacement scans at fixed angles we can also compare angular scans at fixed displacements. In Supplementary figure 11 we compare the angular dependency of the calculated relative energetic stability with the corresponding atom-based CS scores for four displacements giving the minimum energy structures for the anatase (101)/(001) interface (see figures 2 and 4 in the main text). Both Supplementary figures 10 and 11 show that the atom-based CS scores do not correspond well to the calculated relative energetic stabilities.
