# Supplementary material for: An unconstrained approach to systematic structural and energetic screening of materials interfaces
Source: Nat Commun. 2022 Oct 20;13:6236. doi: 10.1038/s41467-022-33414-6 (PMC9585034; doi:10.1038/s41467-022-33414-6)
Supplement: Supplementary file 4 — Description of Additional Supplementary Files [file 41467_2022_33414_MOESM4_ESM.docx]

**Description of Additional Supplementary Files**

File Name: Supplementary Data 1

Description: Disk Interface Model preparation code and examples
